# IL1A: a novel prognostic biomarker and potential therapeutic target for renal clear cell carcinoma

**DOI:** 10.32604/or.2025.061978

**Published:** 2025-06-26

**Authors:** JI ZENG, XUETENG MENG, YUAN ZHANG, JUN LI, TAOTAO MA, CHENG HUANG

**Affiliations:** 1 Inflammation and Immune Mediated Diseases Laboratory of Anhui Province, Anhui Institute of Innovative Drugs, School of Pharmacy, Anhui Medical University, Hefei, 230032, China; 2 Department of Pharmacy, Ma’anshan City Hospital of Traditional Chinese Medicine, Ma’anshan, 243000, China; 3 Anhui Provincial Institute of Translation Medicine, Anhui Medical University, Hefei, 230032, China; 4 The Third Affiliated Hospital of Anhui Medical University, The First People’s Hospital of Hefei, Hefei, 230032, China

**Keywords:** Biomarker, Inflammation, Interleukin, Prognosis, Renal cell carcinoma, Tumorigenesis

## Abstract

**Background:**

Renal cell carcinoma (RCC) is a prevalent malignancy characterized by a rising incidence and significant mortality. Interleukins (ILs) are crucial in regulating immune cell trafficking and exhibit anti-tumor properties. However, limited research has explored the expression levels and prognostic significance of interleukins in RCC.

**Methods:**

In this comprehensive study, we performed a detailed analysis of interleukins in RCC patients using multiple bioinformatics tools, including Oncomine, UALCAN, GEPIA, Kaplan-Meier plotter, cBioPortal, GeneMANIA, TRRUST, STRING, and Linked Omics.

**Results:**

Our analysis demonstrated a significant upregulation in the transcriptional levels of IL4, IL7, IL15, IL16, IL23A, IL26, and IL32 were significantly upregulated in RCC tissues, indicating their potential involvement in the pathogenesis of this malignancy. In contrast, IL1A, IL11, and IL27 were downregulated, indicating their potential function as tumor suppressors. Significant correlations were identified between the expression levels of IL11, IL23A, IL27, IL32, and the pathological stage of RCC patients. The expression levels of IL1A, IL4, IL11, IL15, IL16, IL23A, IL26, IL27, and IL32 were significantly correlated with improved prognosis. The differentially expressed interleukins primarily function in cytokine-cytokine receptor interactions and immune response-regulating signaling pathways. homeobox A10 (HOXA10), v-myb myeloblastosis viral oncogene homolog (avian) (MYB), v-rel reticuloendotheliosis viral oncogene homolog A (avian) (RELA), and nuclear factor of kappa light polypeptide gene enhancer in B-cells 1(NFKB1) are key transcription factors for ILs, while LCK proto-oncogene (LCK), LYN proto-oncogene (LYN), spleen associated tyrosine kinase (SYK), Janus kinase 3 (JAK3), and FER tyrosine kinase (FER) are IL targets. IL expression significantly correlated with the infiltration of six distinct immune cell types. IL1A potentially exerts an anti-tumor effect in RCC prognosis by inducing neutrophil extracellular traps (NETs). Additionally, NFKB1 may positively regulate IL1A, providing a rationale for further *in vivo* and clinical studies.

**Conclusion:**

In conclusion, our study demonstrates the potential role of IL 1A in the prognosis of RCC and establishes a theoretical foundation for subsequent *in vivo* and clinical investigations.

## Introduction

Renal cell carcinoma (RCC), the most common type of kidney cancer, accounts for approximately 400,000 new cases and 175,000 deaths annually. The incidence of RCC has shown a consistent annual increase RCC is classified into three primary histological subtypes: clear cell RCC (ccRCC), papillary RCC (pRCC), and chromophobe RCC (chRCC). ccRCC, which represents nearly 75% of RCC cases, is the most prevalent pathological subtype [1]. This subtype originates from the proximal convoluted tubule and frequently exhibits aggressive biological behavior. Among renal cell carcinoma subtypes, kidney renal clear cell carcinoma (KIRC) is associated with the poorest prognosis [2]. Kidney cancer, including RCC, demonstrates promising responses to immunotherapy. As a result, novel therapeutic strategies, such as kinase inhibitors, are currently being explored. Currently, surgical resection is the primary treatment for ccRCC, while existing pharmacological therapies are associated with substantial side effects and limited efficacy. Given the limitations in the current literature, there is an urgent need to identify effective therapeutic biomarkers to improve diagnostic accuracy and predict clinical outcomes more reliably [3].

Interleukins (ILs), a subset of cytokines, are pivotal for mediating immune cell communication in response to specific antigens. Cytokines are secreted by diverse cell types, including macrophages, T-lymphocytes, mast cells, stromal cells, epithelial cells, and tumor cells. The involvement of interleukins in various tumor-associated mechanisms has been extensively studied, resulting in their application in cancer therapeutic strategies.

Although the expression profiles and functions of certain interleukins in KIRC are generally understood, their precise roles as therapeutic targets and prognostic biomarkers remain unclear and present a significant challenge [4]. Recent advancements in databases and gene sequencing technologies have made comprehensive interleukin analysis more feasible. Through in-depth bioinformatics analysis and partial experimental validation, we identified a potential role of IL1A in KIRC prognosis. Our findings aim to assist clinicians in selecting suitable therapeutic drugs for KIRC patients.

## Databases and Methods

**ONCOMINE** (**www.oncomine.org**) (accessed on 04 March 2025)

The student’s *t*-test was employed to analyze interleukin expression levels in RCC and normal kidney tissues. Statistical significance was defined as *p* < 0.05, with a fold change threshold of 2.0. Genes were ranked within the top 10%, and the analysis focused exclusively on mRNA data.

**UALCAN** (**http://ualcan.path.uab.edu/analysis.html**) (accessed on 04 March 2025)

Using the UALCAN database, we selected KIRC from the TCGA datasets and input the following interleukins: IL1A IL4, IL7, IL11, IL15, IL16, IL23A, IL26, IL27, IL32. We then clicked ‘explore’ and selected ‘expression analysis’ to obtain interleukin expression data. A Student’s *t*-test was performed for statistical analysis, with a significance threshold set at *p* < 0.05.

**GEPIA** (**http://gepia.cancer-pku.cn/index.html**) (accessed on 04 March 2025)

Using the GEPIA online tool, we selected the Boxplots module and entered IL1A, IL7, IL11, IL15, IL16, IL23A, IL26, IL27, and IL32. choose KIRC dataset, The KIRC dataset was chosen, with TCGA normal and GTEx data selected for Matched Normal. The plot function was then used to visualize the expression differences of these interleukins in KIRC and normal tissues. In the Multiple Gene Analysis module, the same interleukins were entered. The KIRC dataset was selected, with Only Tumor data chosen for Matched Normal data. The plot function was then executed to perform a multi-gene comparison. Within the Stage plot module, each interleukin was individually entered. The KIRC dataset was selected, and the plot function was used to analyze their expression patterns across various pathological stages. Statistical significance was assessed using Student’s *t*-test, with a threshold of *p* < 0.05.

**Kaplan-Meier plotter analysis** (**https://www.kmplot.com/**) (accessed on 04 March 2025)

Utilizing the Kaplan-Meier Plotter database, we input IL1A, IL4, IL7 IL11, IL15, IL16, IL23A, IL26, IL27, IL32 under the pan-cancer module. We selected the KIRC dataset (n = 530), and analyzed Overall Survival (OS) (n = 7462) and Recurrence-Free Survival (RFS) (n = 4420) to generate survival curves, hazard ratio (HR),95% confidence intervals (CI), and log-rank *p*-values. Results were considered statistically significant if *p* < 0.05.

**cBioPortal** (**www.cbioportal.org**) (accessed on 04 March 2025)

We utilized the cBioPortal database to investigate the relationship between interleukin gene alterations and overall survival (OS), disease-specific survival (DSS), progression-free survival (PFS), and disease-free survival (DFS) of patients with renal cell carcinoma. Using the KIRC (TCGA, Pan Cancer Atlas) dataset (n = 512), we queried mRNA and protein expression z-scores for IL1A, IL4, IL7, IL11, IL15, IL16, IL23A, IL26, IL27, and IL32. Kaplan-Meier survival curves were constructed for OS, DSS, PFS, and DFS, with statistical significance was determined using the log-rank test *(p* < 0.05).

**STRING** (**https://string-db.org/**) (accessed on 04 March 2025)

Using the STRING database, we employed the multiple proteins module to input interleukin genes, including IL1A, IL4, IL7, IL11, IL15, IL16, IL23A, IL26, IL27, and IL32. We selected “*Homo sapiens*” as the species to construct protein-protein interaction (PPI) networks for interleukins and analyze their interactions.

**GeneMANIA analysis** (**http://www.genemania.org/**) (accessed on 04 March 2025)

GeneMANIA was utilized to construct interleukin gene co-expression and pathway networks and to predict their potential functions.

**Linked Omics** (**http://www.linkedomics.org/**) (accessed on 04 March 2025)

We utilized the Linked Omics database to select the TCGA_KIRC HisSeq RNA dataset, specifically focusing on the kidney clear cell renal carcinoma sample set (n = 537). The following interleukins were analyzed: IL1A, IL4, IL7, IL11, IL15, IL16, IL23A, IL26, IL27, and IL32. Pearson Correlation tests were performed to generate volcano plots and heat maps for visual analysis. Gene Set Enrichment Analysis (GSEA) was conducted to examine biological processes, KEGG pathways, kinase targets, and transcription factor targets. Weighted set coverage was applied to minimize duplication, with a false discovery rate (FDR) <0.05, a minimum of 3 genes, and 500 simulations.

**GEO DATA** (**https://www.ncbi.nlm.nih.gov/geo/**) (accessed on 04 March 2025)

Access the GEO database homepage and search using the keywords “renal cell carcinoma” or “KIRC”. The screening criteria were as follows:1. Both control and experimental groups must be included; 2. A minimum sample size (n) of 20; 3. Availability of GEO Platform (GPL) annotation files with Gene symbols. The following datasets were downloaded: GSE6344, GSE15641, GSE16441, GSE16449, GSE53757, GSE71963. GPL6480 was utilized for GSE71963, GSE16441, and GSE16449. GPL570 was employed for GSE53757 and GSE36895. The chip platform for GSE15641 and GSE6344 was GPL96.

**TRRUST** (**https://www.grnpedia.org/trrust/**) (accessed on 04 March 2025)

To investigate the regulatory interactions between transcription factors (TFs) and their targets, a dataset encompassing 800 human TFs was analyzed. The TRRUST IL1A module’s search function enables the identification of transcription factors regulating interleukin targets, including IL4, IL7, IL11, IL15, IL16, IL23A, IL26, IL27, and IL32.

**TIMER** (**https://cistrome.shinyapps.io/timer/**) (accessed on 04 March 2025)

Access the TIMER database, proceed to the Immune Association module, and select the Gene tab. Enter the following interleukin genes sequentially: IL1A, IL4, IL7, IL11, IL15, IL16, IL23A, IL26, IL27, and IL32. Evaluate the association between interleukin levels and immune cell infiltration in kidney renal clear cell carcinoma (KIRC, n = 533). Utilize the Survival module to examine the correlation between clinical outcomes, immune cell infiltration, and the expression of the selected interleukins.

## Experimental Materials and Methods

### Reagents and antibody

The IL-1α protein was obtained from MedChemExpress (MCE, HY-P7027; Shanghai, China). Antibodies against IL-1α, Citrullinated Histone H3 (Cit-H3), matrix metalloproteinase9 (MMP9), and proliferating cell nuclear antigen (PCNA) were sourced from Abcam (Cambridge, UK; catalog numbers: ab300501; ab5103; ab137867; ab92729). The Goat anti-rabbit/mouse IgG (HRP-conjugated) secondary antibody was procured from Abmart (Shanghai, China; catalog number: ab205718). Antibodies targeting β-actin and MMP-2 antibodies were acquired from Zenbio (Chengdu, China; catalog numbers: 250136;380817). Small interfering RNA (siRNA) targeting NFκB1 was purchased from Hanbio Biotechnology (Shanghai, China). All primers were sourced from General Biology (Chuzhou, China).

### Cell culture

Caki-1 cells were obtained from Fu Heng Biology and cultured in Dulbecco’s Modified Eagle’s Medium (DMEM) (Shanghai Darthill Biotechnology Co. Ltd., LOT number 2335274, Shanghai, China) supplemented with 10% Fetal Bovine Serum (FBS) (Bio-Channel Biotechnology Co. Ltd., catalog number BC-SE-FBS08, Nanjing, China). HK-2 cells were also sourced from Fu Heng Biology and maintained in RPMI Medium 1640 (1X) (ThermoFisher Biochemical Products (Beijing, China) Co. Ltd., LOT number 8123408) supplemented with 10% FBS. Both cell lines were incubated at 37°C in a humidified atmosphere containing 5% CO_2_.

### Cell transfection

The following siRNAs were designed and synthesized by Hippo (Huzhou, China): siNFKB1(s)5′-GGAGACAUCCUUCCGCAAAdTdT-3′(as)5′UUUGCGGAAGGAUGUCUCCdTdT-3′.siCtrl(s)5′-UUCUCCGAACGUGUCACGUdTdT-3′(as)5′-ACGUGACACGUUCGGAGAAdTdT-3′.Cells were seeded in 6-well plates one day before transfection to reach 60%–80% confluence. For transfection, 10 μL of NFKB1-targeting siRNA (Anhui Emosa Biotechnology Co. Ltd., Hefei, China) and 10 μL of Advanced series high-efficiency DNA/RNA transfection reagent (Zeta-Life Catalog No.AD600025, San Francisco, CA, USA) were mixed and incubated at room temperature for 15 min. The nucleic acid complexes were added to each well and gently mixed. After 24 h of transfection, cells were cultured under standard conditions, and RNA was extracted 48 h later. mRNA expression was subsequently analyzed. Silencing efficiency was evaluated by qPCR analysis of mRNA levels.

### Cell wound healing assay

Following a series of standardized treatments, trypsinized cells were seeded in 6-well plates containing complete medium at a density of approximately 1 × 10^5^ cells per well. Upon reaching 75% confluence, the cells were scratched using a sterile pipette tip and subsequently washed with PBS to eliminate cellular debris. Subsequently, serum-free medium was added to the 6-well plateS, which were then incubator for 48 h. Throughout the incubation period, cells progressively migrated into the wound area, a process referred to as cell wound healing. This migration process was captured using an inverted fluorescence microscope (Primovert, Carl ZEISS, Shanghai, China), and the wound closure rate was quantified.

### Cell transwell migration assay

The Transwell assay utilizes a 24-well Boyden chamber (Corning, New York, NY, USA) equipped with an 8.0 μm pore size polycarbonate membrane. For the migration assay, trypsinized cells were seeded in the upper chamber with 100 μL of serum-free medium (seeding density approximately 1 × 10^5^ cells/well). Subsequently, 600 μL of complete medium, serving as a chemoattractant, was added to the lower chamber. After 24 h of incubation at 37°C, residual cells on the upper surface of the membrane were removed using cotton swabs. The membrane was then fixed with 4% paraformaldehyde, stained with crystal violet solution, and photographed using an inverted fluorescence microscope (Primovert, Carl ZEISS).

### Neutrophil extraction and formation of NETs

All experimental animals were procured from the Laboratory Animal Center of Anhui Medical University. Mice were euthanized in compliance with approved protocols (The experimental protocol received approval from the Animal Ethics Committee of Anhui Medical University, Approval No.: LISC20150277), and femurs and tibiae were aseptically collected and placed in sterile Petri dishes. The epiphyses were excised, and the bone marrow cavities were flushed with complete medium (RPMI-1640 supplemented with 10% FBS and 1% penicillin-streptomycin, Gibco, Catalog No. 15140122, Grand Island, UK). The resulting suspension was filtered through a sterile 100-micron nylon mesh. Subsequently, the cell isolation solution from the mouse neutrophil isolation kit (Solarbio, Catalog No. P9201, Beijing, China) was carefully layered into centrifuge tubes, followed by gentle overlaying of the cell suspension on top. The tubes were then centrifuged at 1000× *g* for 30 min at room temperature. The neutrophil-containing layer was aspirated and transferred to a new tube containing 10 mL of PBS. The mixture was then centrifuged. Following two additional washes with PBS, the cells were resuspended in RPMI-1640.

### q-PCR

Total RNA was collected from cells using TRIzol reagent (Invitrogen, Catalog No. 15596026, Waltham, MA, USA). First strand cDNA was synthesized using the Thermoscript RT-PCR synthesis kit (Invitrogen, Catalog No. 12574026, CA, USA) according to the manufacturer’s instructions. mRNA was analyzed using the Thermoscript RT-qPCR kit in the ABI Prizm First Step Plus Real-Time Fluorescent Quantitative PCR System

(Applied Biosystems, Foster City, CA, USA) for real-time quantitative PCR analysis of mRNA. These products were used as templates for amplification using SYBR Green PCR amplification reagents (Thermo Fisher, Catalog No. 4309155, Waltham, MA, USA) and gene-specific primers. Relative expression levels were calculated according to the standard 2^−ΔΔCt^ method. β-actin served as the reference gene for normalization during the analysis. The specific primers were designed to amplify the genes encoding IL1A, IL7, IL11, IL15, IL16, IL23A, IL26, IL27, IL32, LCK, LYN, SYK, JAK3, FER, NFKB1, PCNA, MMP2, MMP9 as well as β-actin. The sequences of all primers used in the article are listed below (Table 1).

**Table 1:** Sequences of all primers

Target genes(mouse)	Primer sequences	
β-actin	Forward	CATGTACGTTGCTATCCAGGC
	Reverse	CTCCTTAATGTCACGCACGAT
IL1A	Forward	TGGTAGTAGCAACCAACGGGA
	Reverse	ACTTTGATTGAGGGCGTCATTC
IL7	Forward	TTGGACTTCCTCCCCTGATCC
	Reverse	TCGATGCTGACCATTAGAACAC
IL11	Forward	CGAGCGGACCTACTGTCCTA
	Reverse	GCCCAGTCAAGTGTCAGGTG
IL15	Forward	TTGGGAACCATAGATTTGTGCAG
	Reverse	GGGTGAACATCACTTTCCGTAT
IL16	Forward	GCCGAAGACCCTTGGGTTAG
	Reverse	GCTGGCATTGGGCTGTAGA
IL23A	Forward	CTCAGGGACAACAGTCAGTTC
	Reverse	ACAGGGCTATCAGGGAGCA
IL26	Forward	GCTGTTAGTCACTCTGTCTCTTG
	Reverse	GGACAATGTTCCCCTTGGGTA
IL27	Forward	ACCGCTTTGCGGAATCTCA
	Reverse	AGGTCAGGGAAACATCAGGGA
IL32	Forward	TGGCGGCTTATTATGAGGAGC
	Reverse	CTCGGCACCGTAATCCATCTC
LCK	Forward	TGCCATTATCCCATAGTCCCA
	Reverse	GAGCCTTCGTAGGTAACCAGT
LYN	Forward	GCTTTTGGCACCAGGAAATAGC
	Reverse	TCATGTCGCTGATACAGGGAA
SYK	Forward	CATGGAAAAATCTCTCGGGAAGA
	Reverse	GTCGATGCGATAGTGCAGCA
JAK3	Forward	TTCGGGCTACGCAAGGATTTG
	Reverse	AGGCTGAGACACTCACCCT
FER	Forward	ACTCTGGACCTTTACACAGGC
	Reverse	GCTTTGTCGTATCGTTCCTTGG
NFKB1	Forward	AACAGAGAGGATTTCGTTTCCG
	Reverse	TTTGACCTGAGGGTAAGACTTCT
PCNA	Forward	CCTGCTGGGATATTAGCTCCA
	Reverse	CAGCGGTAGGTGTCGAAGC
MMP2	Forward	TACAGGATCATTGGCTACACACC
	Reverse	GGTCACATCGCTCCAGACT
MMP9	Forward	TGTACCGCTATGGTTACACTCG
	Reverse	GGCAGGGACAGTTGCTTCT

### Western blot

Proteins (30 or 50 mg) were extracted from the aforementioned cell lines above and kidney tissues using RIPA lysis buffer (Beyotime, Catalog No. P0013B, Beijing, China) supplemented with 1% phenyl methyl sulfonyl fluoride (PMSF; Beyotime, Catalog No. ST506, Shanghai, China). Protein concentration was subsequently measured using the BCA Protein Detection Kit (Beyotime, Catalog No. P0012, Beijing, China). The membrane was blocked with 5% skim milk. Following a 1.5-h incubation, the membrane was washed three times with Tris-buffered saline containing 0.1% Tween (TBST), each wash lasting 10 min. All procedures were conducted at room temperature. Subsequently, whole extracts were separated using 10% or 12.5% sodium dodecyl sulfate polyacrylamide gel electrophoresis (SDS-PAGE), transferred to a polyvinylidene difluoride membrane (Millipore, headquartered in Boston, USA, IPVH00010), which were incubated with primary antibodies against IL1A (1:1000), Cit-H3 (1:1000), MMP9 (1:1000), MMP2 (1:500), PCNA (1:1000) and β-actin (1:1000). The membranes were then washed in TBS-Tween 20 and incubated with secondary antibodies at a dilution of 1:500. After extensive washing in TBS-Tween 20, protein bands were visualized using an enhanced chemiluminescence (ECL) system (ECL-plus, ThermoFisher Scientific, Pittsburgh, PA, USA).

### Immunofluorescence staining

Neutrophil was fixed for 30 min with 4% paraformaldehyde and then permeabilized with 0.1% Triton X-100 for 20 min after washing with phosphate-buffered saline (PBS). Samples were incubated with blocking buffer (5% BSA or normal serum in PBS) for 1 h at room temperature to prevent nonspecific binding. Following the addition of the anti-IL1A primary antibody (1:200), the samples are incubated at 37°C for 45 min. After incubation, the samples were washed three times with PBS for 5 min each at room temperature. The secondary antibody (1:50; Abcam, Catalog No. ab205718, Cambridge, UK) was added, and the cells were incubated at room temperature for 45 min. After incubation, the samples were washed again as described above. DAPI (Beyotime, C1005) was added for 10 min to stain the cells. At last, images were captured using a live cell imager after the addition of immunofluorescence blocking solution.

### Statistical analysis

Experimental data are presented as mean± standard error (Mean ± SEM) of the mean (SEM) and analyzed using GraphPad Prism 9.0 software. Statistical significance was defined as *p* < 0.05. Protein band intensity was quantified using ImageJ software.

## Results

### Aberrant expression of interleukin in patients with KIRC

**Figure 1:**
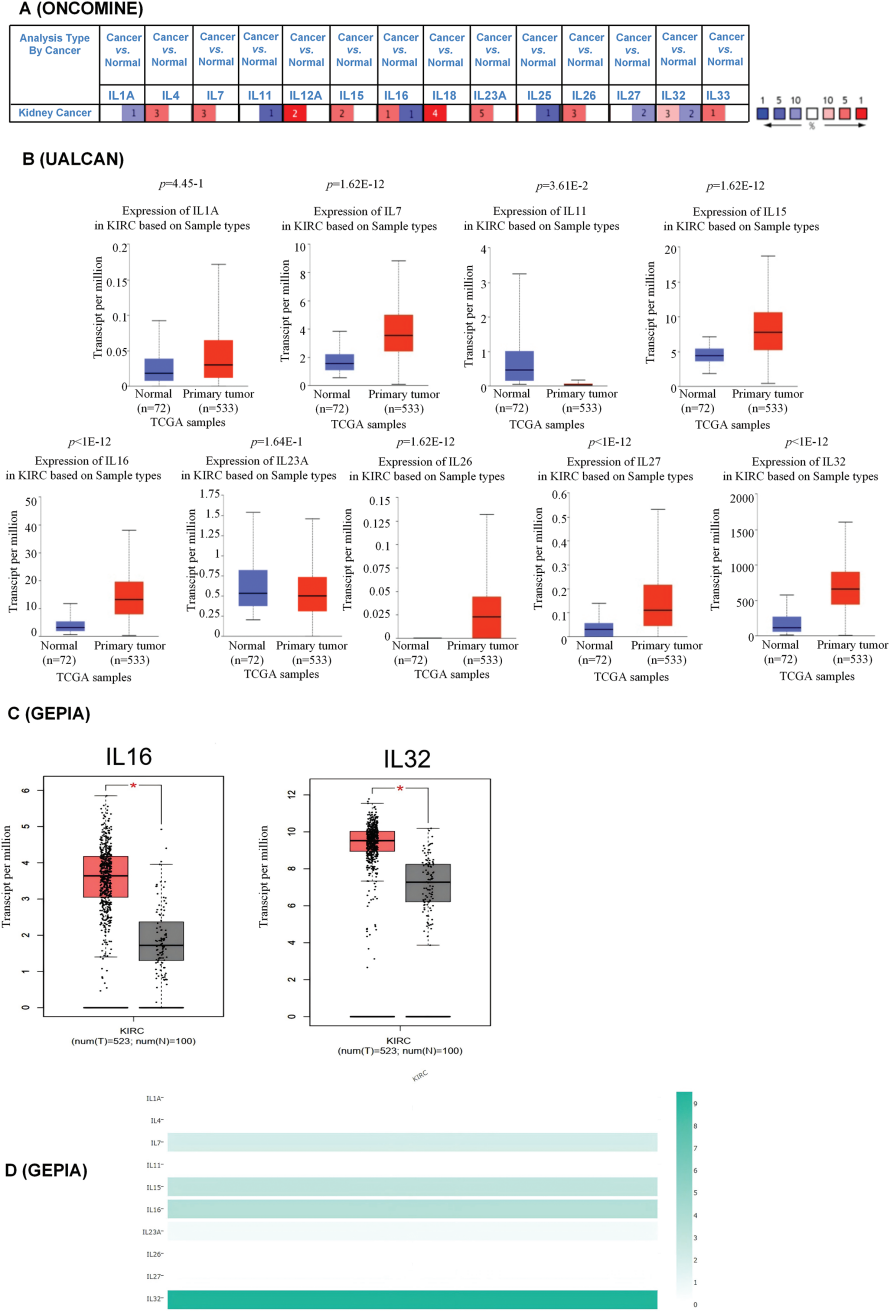
Aberrant expression of interleukin in patients with RCC. (A) mRNA expression of interleukin in RCC (ONCOMINE). Color is determined by the highest gene rank percentile gene based on log fold change; red represents upregulation and blue represents downregulation. (B) mRNA expression of interleukin in RCC tissues and normal kidney tissues (UALCAN). (C) mRNA expression of interleukin in RCC tissues and normal kidney tissues (GEPIA). *p < 0.05. (D) The relative level of interleukin in RCC.

Using ONCOMINE analysis, we first examined the mRNA transcriptional levels of interleukins in KIRC tissues and normal kidney tissues. The results revealed significant upregulation of IL4, IL7, IL15, IL16, IL23A, IL26, and IL32 in KIRC tissues, whereas IL1A, IL11, and IL27 were significantly downregulated (Fig. 1A and Table 2). Next, we evaluated interleukin expression levels in KIRC using RNA sequencing data from the TCGA database via the UALCAN website. As shown in Fig. 1B, the mRNA expressions of IL7 (*p* = 1.62E − 12), IL15 (*p* = 1.62E − 12), IL16 (*p* < 1E − 12), IL26 (*p* = 1.62E − 12), IL27 (*p* < 1E − 12), and IL32 (*p* < 1E − 12) were upregulated in KIRC tissues, whereas the level of IL11 (*p* = 3.61E − 2) was downregulated. Additionally, we analyzed interleukin transcriptional levels using GEPIA. The mRNA expression levels of IL16 and IL32 were significantly elevated in 523 KIRC cases compared to 100 cases of normal controls (Fig. 1C). We further analyzed the relative expression levels of interleukins in KIRC tissues. Among all interleukins, IL32 exhibited the highest expression level (Fig. 1D). These findings suggest that the IL16 and IL32 levels are significantly associated with KIRC. We performed a gene expression analysis of interleukins (ILs) utilizing multiple datasets from the GEO database. Our analysis revealed a downregulation of IL1A in KIRC, while IL4, IL15, IL16, IL26, and IL27 were upregulated. IL7, IL11, and IL32 displayed mixed regulation patterns, including both upregulation and downregulation. Moreover, the expression levels of ILs in some datasets lacked statistical significance (*p* > 0.05), contrasting with the TCGA database analysis results (Fig. S1A–F).

**Table 2:** The mRNA levels of interleukin in different types of RCC tissues and normal renal tissues at transcriptome level (ONCOMINE)

TLR	Type	Fold change	*p*-value	*t*-test
IL1A	Clear cell renal cell carcinoma	−2.936	1.36E − 14	−13.778
IL4	Clear cell renal cell carcinoma	2.151	1.69E − 12	9.527
IL7	Hereditary clear cell renal cell carcinoma	2.447	3.24E − 11	10.23
	Non-hereditary clear cell renal cell carcinoma	2.37	8.91E − 09	7.204
	Clear cell renal cell carcinoma	3.129	6.82E − 07	7.109
IL11	Clear cell renal cell carcinoma	−3.505	2.54E − 04	−4.545
IL15	Hereditary clear cell renal cell carcinoma	2.123	1.34E − 11	9.537
IL16	Clear cell renal cell carcinoma	2.646	6.9E − 5	6.428
IL23A	Clear cell renal cell carcinoma	20.735	3.56E − 7	7.523
	Hereditary clear cell renal cell carcinoma	9.828	4.94E − 8	8.261
	Non-hereditary clear cell renal cell carcinoma	6.675	1.12E − 6	5.965
	Clear cell renal cell carcinoma	2.941	8.11E − 4	3.978
IL26	Clear cell renal cell carcinoma	4.458	2.98E − 16	13.222
IL27	Clear cell renal cell carcinoma	−3.139	0.003	−3.439
IL32	Clear cell renal cell carcinoma	4.785	3.58E − 5	6.207
	Non-hereditary clear cell renal cell carcinoma	4.479	6.25E − 06	7.194

### Clinicopathological characteristics of interleukin in patients with KIRC

**Figure 2:**
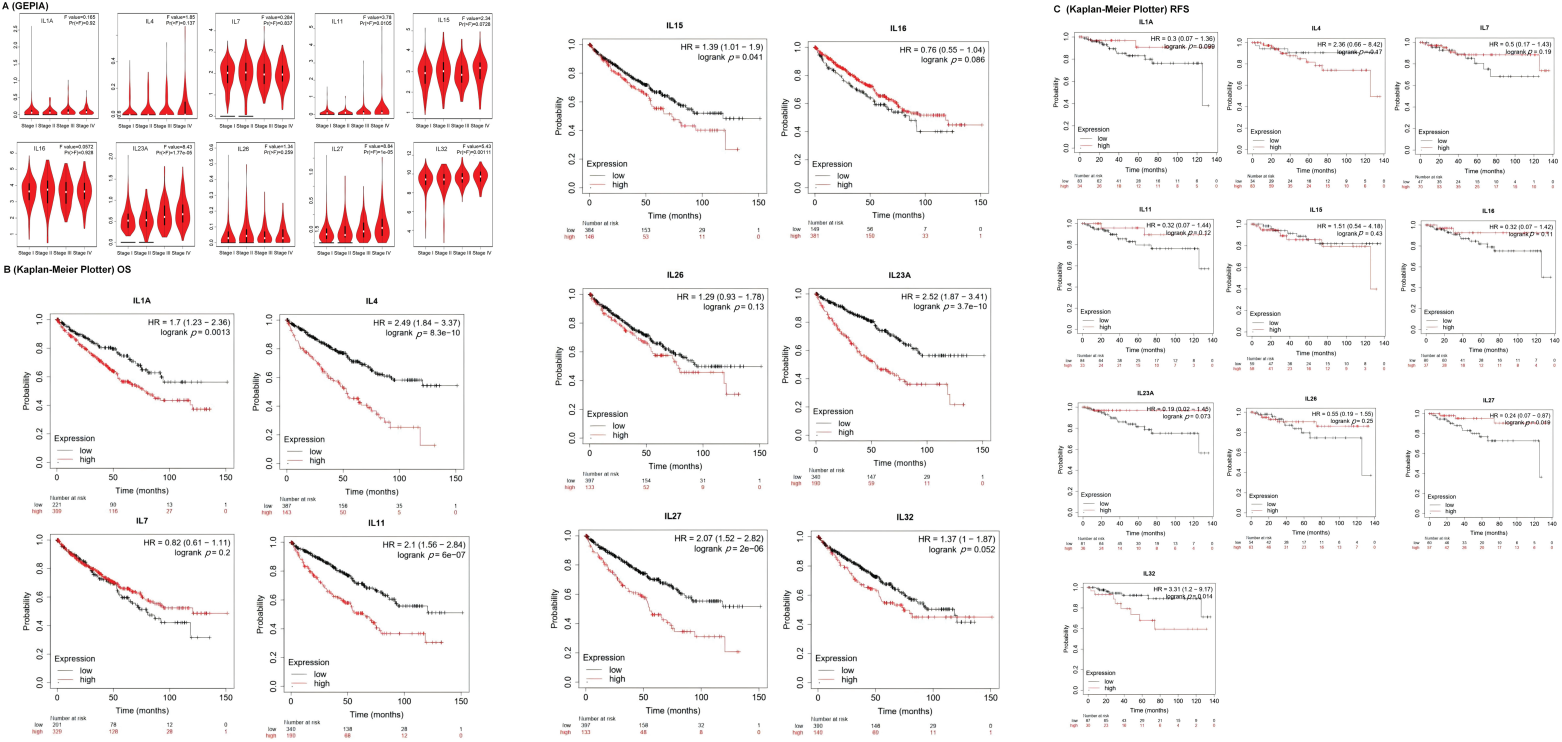
The clinicopathological characteristics of interleukin in patients with RCC. (A) Correlation between mRNA expression of interleukin and tumor stages of RCC patients (GEPIA). (B) Relationship between interleukin expression and OS in RCC patients (Kaplan-Meier Plotter). (C) Relationship between interleukin expression and RFS in RCC patients (Kaplan-Meier Plotter).

**Figure 3:**
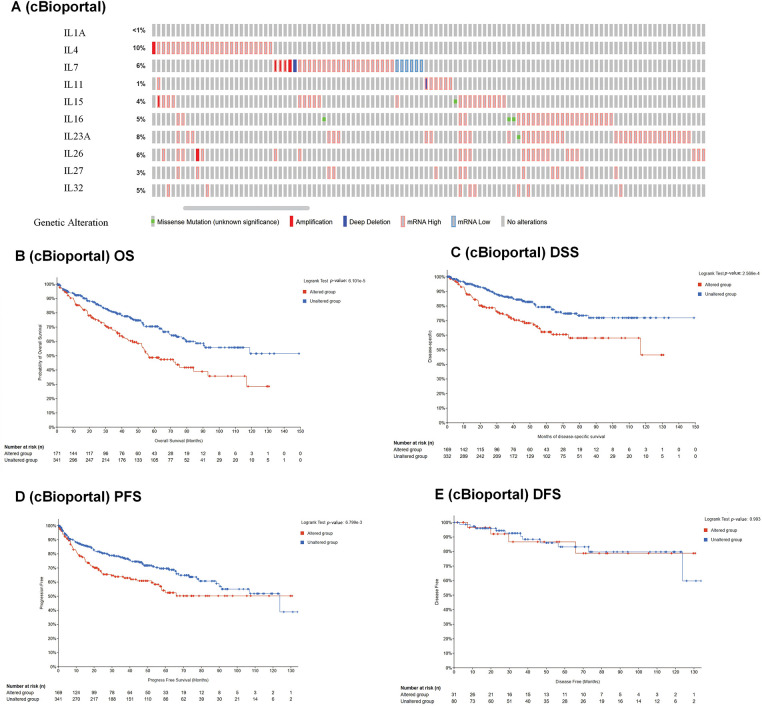
Genetic alteration and prognostic value analyses of interleukin in RCC patients. (A) Genetic alteration of interleukin in RCC patients (cBioporta). (B) The relationship between genetic alteration and OS (cBioporta). (C) The relationship between genetic alteration and DSS (cBioporta). (D) The relationship between genetic alteration and PFS (cBioporta). (E) The relationship between genetic alteration and DFS (cBioporta).

To investigate the association between interleukins and the clinicopathological characteristics of KIRC, we employed the GEPIA database. As depicted in Fig. 2A, IL11 (*p* = 0.010), IL23A (*p* = 1.77e − 05), IL27 (*p* = 1e − 05) and IL32 (*p* = 0.001) exhibited significant correlations with tumor stage. Subsequently, we assessed the prognostic significance of interleukins using the Kaplan-Meier Plotter database. As illustrated in Fig. 2B, IL1A (*p* = 0.0013), IL4 (*p* = 8.3e − 10), IL11 (*p* = 6e − 07), IL15 (*p* = 0.041), IL23A (*p* = 3.7e − 10) and IL27 (*p* = 2e − 06) demonstrated significant associations with overall survival (OS). Additionally, IL27 (*p* = 0.019), and IL32 (*p* = 0.014) showed significant associations with relapse-free survival (RFS) (Fig. 2C). To analyze genetic alterations in interleukins, we examined their associations with overall survival (OS) and disease-specific survival (DSS) in KIRC patients using cBioportal. Fig. 3A illustrates the alteration frequencies of IL1A (<1%), IL4 (10%), IL7 (6%), IL11 (1%), IL15 (4%), IL16 (5%), IL23A (8%), IL26 (6%), IL27 (3%), and IL32 (5%) in the queried KIRC samples. Additionally, cBioportal analysis demonstrated significant associations between genetic alterations and OS (*p* = 6. 101e − 5) (Fig. 3B), DSS (*p* = 2.569e − 4) (Fig. 3C), PFS (*p* = 6.799e − 3) and DFS (*p* = 0.993) (Fig. 3D,E). These results indicate that genetic alterations in interleukins could serve as potential prognostic markers for KIRC patients.

### Co-expression, neighbor gene network, and interaction analyses of interleukin in patients with KIRC

**Figure 4:**
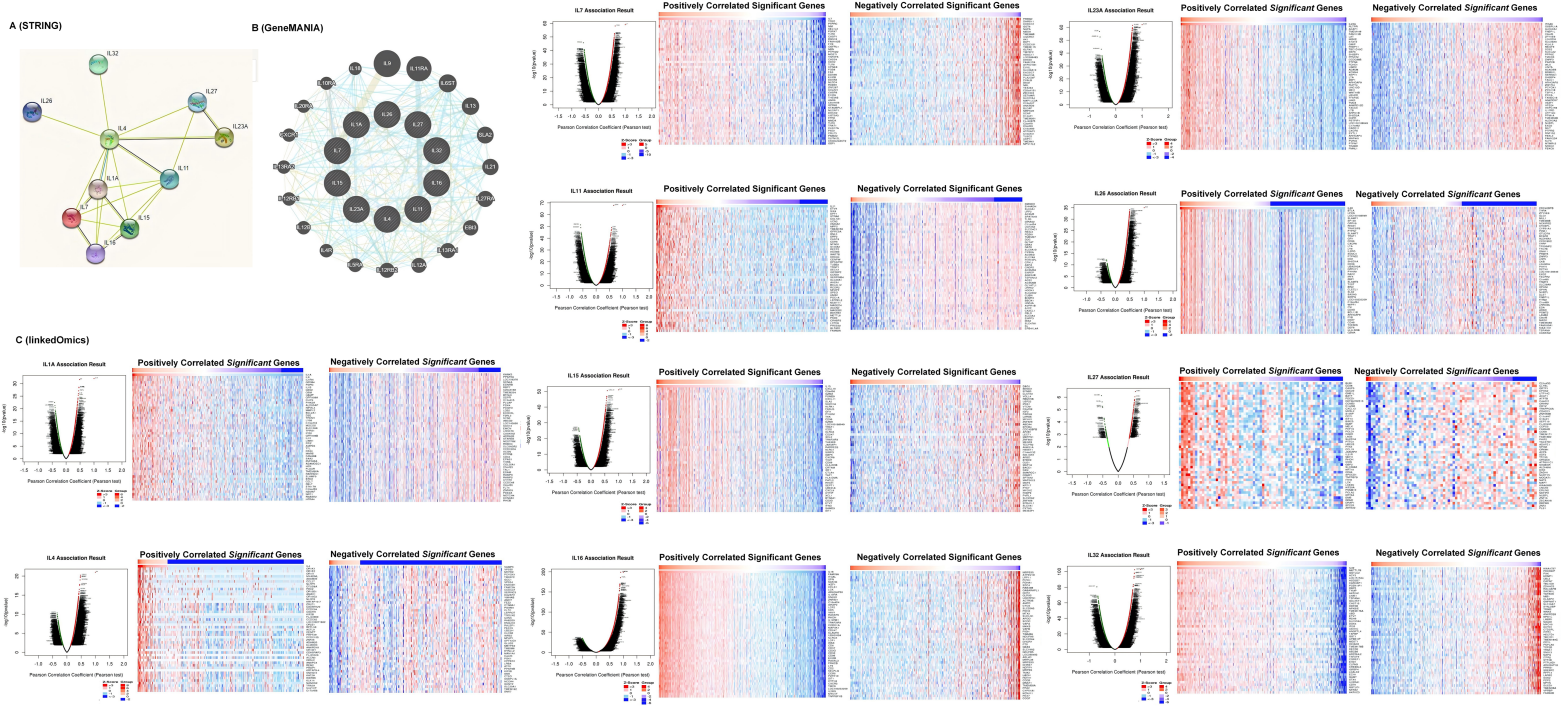
Co-expression, neighbor gene network, and interaction analyses of interleukin in patients with RCC. (A) Protein-protein interaction network of interleukin (STRING). (B) Co-expression network of interleukin (GeneMANIA). (C) Correlations between interleukin and differential expression of genes in RCC, heat maps of top 50 genes positively and negatively correlated with interleukin in RCC (linked Omics).

A protein-protein interaction (PPI) network of interleukins was constructed using STRING to explore potential interactions. As shown in Fig. 4A, the PPI network comprised 10 nodes and 21 edges. To further identify related co-expression genes and their functions, we constructed a gene-gene interactive network using GeneMANIA. This analysis revealed that differentially expressed interleukins are primarily involved in interleukin receptor binding and activity (Fig. 4B). Additionally, Linked Omics was employed to analyze the mRNA sequencing data from 533 KIRC patients in the TCGA database to identify interleukin co-expression genes. The analysis demonstrated significant positive correlations with IL1A, IL4, IL7, IL11, IL15, IL16, IL23A, IL26, IL27, and IL32 genes (red dots), and significant negative correlations with the same genes (green dots) (Pearson’s correlation coefficient > 0.2, FDR < 0.01). Fig. 4C illustrates the top 50 genes with both positive and negative correlations.

### BP, CC, MF, and KEGG pathway analysis of co-expression gene correlated with interleukin in KIRC patients

**Figure 5:**
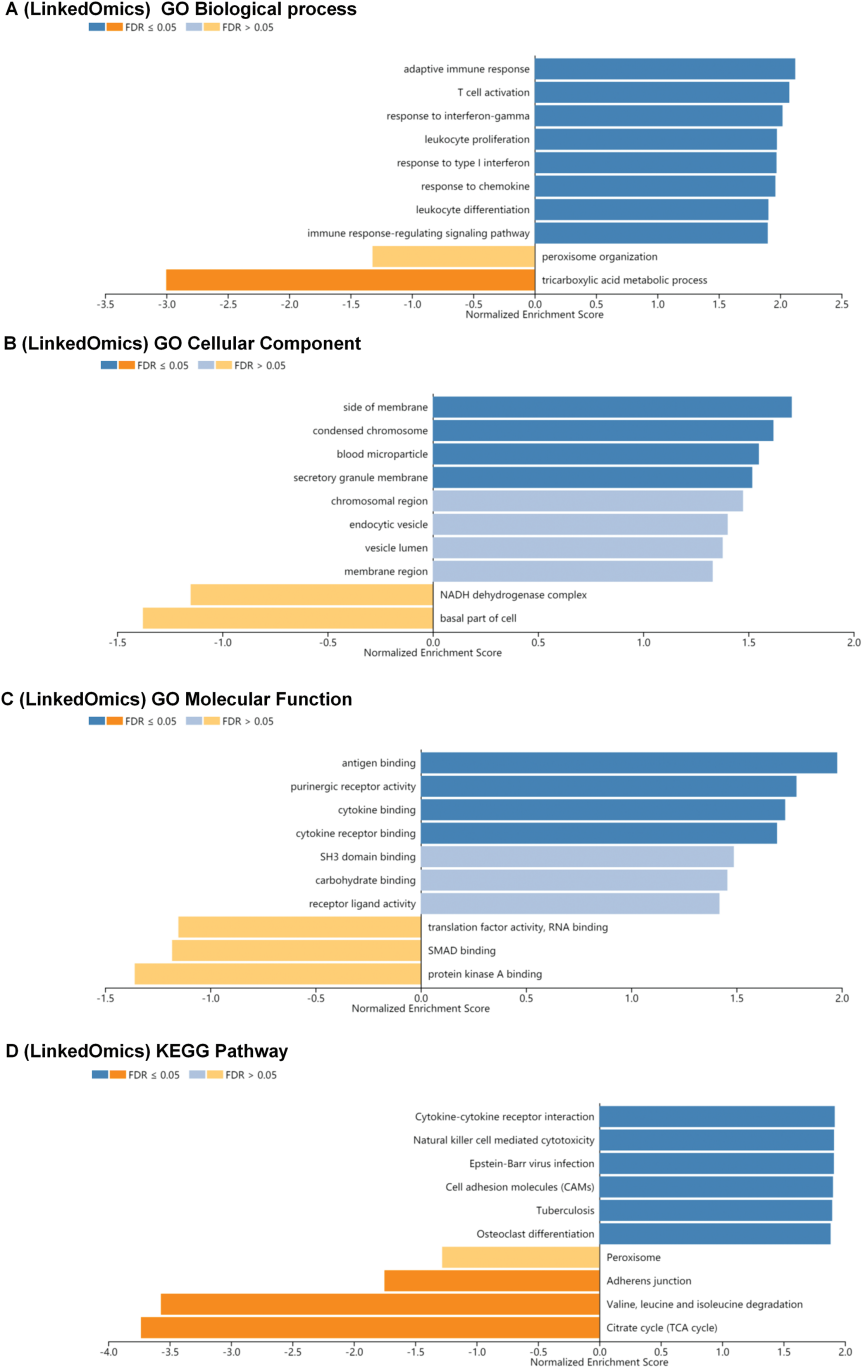
BP, CC, MF and KEGG pathway analysis of co-expression gene correlated with interleukin in RCC patients. (A) The significantly enriched biological processes of interleukin in RCC (linked Omics). (B) The significantly enriched cellular component of interleukin in RCC (linked Omics). (C) The significantly enriched molecular function of interleukin in RCC (linked Omics). (D) KEGG pathway of interleukin in RCC (linked Omics).

We conducted Gene Set Enrichment Analysis (GSEA) to explore Biological Process (BP), Cellular Component (CC), Molecular Function (MF), and KEGG pathway analyses of interleukin-associated genes. In the BP category, pathways such as adaptive immune response, T cell activation, response to interferon-gamma, leukocyte proliferation, response to type I interferon, response to chemokine, leukocyte differentiation, and immune response-regulating signaling were significantly linked to KIRC tumorigenesis and progression (Fig. 5A). In the CC category, the most enriched terms were the side of the membrane condensed chromosome, and the blood microparticle secretory granule membrane (Fig. 5B). MF analysis indicated that differentially expressed interleukins and their neighboring genes were predominantly enriched in functions such as antigen binding, purinergic receptor activity, cytokine binding, and cytokine receptor binding (Fig. 5C). Additionally, KEGG pathway analysis showed significant associations between KIRC tumorigenesis and progression and several pathways, including cytokine-cytokine receptor interaction, natural killer cell mediated cytotoxicity, Epstein-Barr virus infection, cell adhesion molecules (CAMs), tuberculosis, and osteoclast differentiation (Fig. 5D).

### Transcription factor targets, kinase targets of interleukin in KIRC

To explore the interleukin targets in KIRC, we employed GSEA to examine kinase and transcription factor targets within the overlapping co-expression gene set Table 3 lists the top five most significant kinase targets related to interleukins were LCK proto-oncogene, Src family tyrosine kinase (LCK), LYN proto-oncogene, Src family tyrosine kinase (LYN), spleen associated tyrosine kinase (SYK), Janus kinase 3 (JAK3) and FER tyrosine kinase (FER). It was found that LCK, LYN, and JAK3 were significantly upregulated while SYK and FER were significantly downregulated in RCC (Fig. S2A). Additionally, JAK3 and FER genes were significantly associated with overall survival (OS) in KIRC patients (Fig. S2B). Furthermore, using TRRUST, we identified that homeobox A10 (HOXA10), v-myb myeloblastosis viral oncogene homolog (avian) (MYB), v-rel reticuloendotheliosis viral oncogene homolog A (avian) (RELA), and nuclear factor of kappa light polypeptide gene enhancer in B-cells 1 (NFKB1) are involved in interleukin regulation (Table 4). Expression analysis in KIRC showed that NFKB1 and RELA were significantly overexpressed (Fig. S3A). However, only NFKB1 exhibited a significant association with OS and was linked to a favorable prognosis in KIRC patients (Fig. S3B).

**Table 3:** The kinase target of interleukin in RCC (linked Omics)

Gene set	Description	Leading edge number	*p*-value	FDR
Kinase_LCK	LCK proto-oncogene, Src family tyrosine kinase	22	0	0
Kinase_LYN	LYN proto-oncogene, Src family tyrosine kinase	22	0	0
Kinase_SYK	spleen associated tyrosine kinase	15	0	0
Kinase_JAK3	Janus kinase 3	7	0	0.002
Kinase_FER	FER tyrosine kinase	5	0	0.038

**Table 4:** Key regulated factor of interleukin in RCC (TRRUST)

Key TF	Description	Regulated gene	Mode of regulation	*p*-value	FDR
HOXA10	Homeobox A10	IL11	Repression	2.64E − 05	1.32E − 04
		IL15	Activation		
MYB	v-myb myeloblastosis viral oncogene homolog (avian)	IL15	Activation	1.67E − 04	4.17E − 04
		IL7	Activation		
RELA	v-rel reticuloendotheliosis viral oncogene homolog A (avian)	IL1A	Repression	4.44E − 04	5.65E − 04
		IL23A	Activation		
		IL4	Unknown		
NFKB1	Nuclear factor of kappa light polypeptide gene enhancer in B-cells 1	IL1A	Repression	4.52E − 04	5.65E − 04
		IL23A	Activation		
		IL4	Unknown		
JUN	Jun proto-oncogene	IL1A	Unknown	0.00267	0.00267
		IL23A	Unknown		

### Immune cell infiltration of interleukin in patients with KIRC

**Figure 6:**
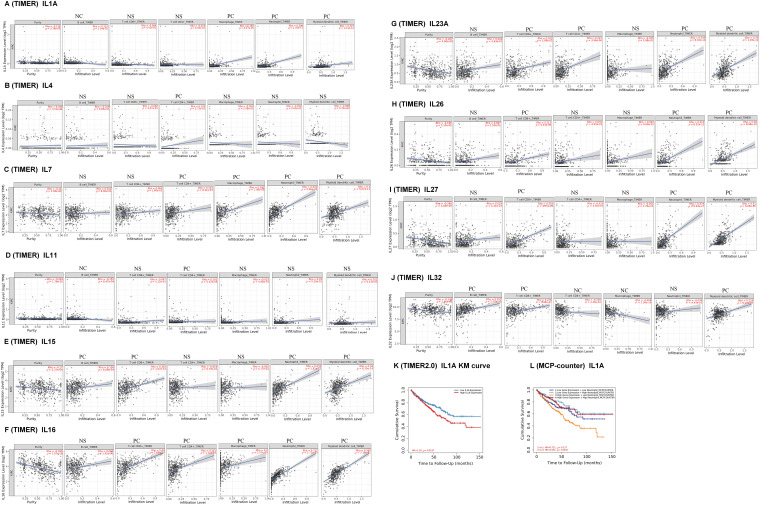
Immune cell infiltration of interleukin in patients with RCC (TIMER). The correlation between the abundance of immune cells and the expression of (A) IL1A, (B) IL4, (C) IL7, (D) IL11, (E)IL15, (F) IL16, (G) IL23A, (H) IL26, (I) IL27 and (J) IL32 in RCC. (K–L) Effect of high neutrophil infiltration on OS in RCC patients with high IL1A expression.

To explore the role of Interleukins in the clinical prognosis of KIRC patients, we examined the correlation between interleukin expression and immune cell infiltration using data from the TIMER database. IL1A expression was found to have a negative correlation with B cells (Cor = −0.115, *p* = 1.34e − 02), and a positive correlation with macrophages (Cor = 0.185, *p* = 6.17e − 05), neutrophils (Cor = 0.308, *p* = 1.35e − 11), and dendritic cells (Cor = 0.323, *p* = 1.21e − 12) (Fig. 6A). IL4 expression was inversely correlated with the infiltration of CD4 + T cells (Cor = 0.103, *p* = 2.73e − 02) (Fig. 6B). IL7 expression positively correlated with the infiltration of CD4+ T cells (Cor = 0.123, *p* = 8.41e − 03), macrophages (Cor = 0.262, *p* = 1.08e − 08), neutrophils (Cor = 0.532, *p* = 5.66e − 35), and dendritic cells (Cor = 0.3, *p* = 5.07e − 11) (Fig. 6C). IL11 expression showed a negative correlation with B cells (Cor = −0.155, *p* = 8.47e − 04) and a positive correlation with CD4+ T cells (Cor = 0.161, *p* = 5.32e − 04) (Fig. 6D). Similar correlations were observed for IL15, IL16, IL23A, IL26, and IL27 (Fig. 6E–I). IL32 expression exhibited a negative correlation with CD4+ T cells (Cor = −0.178, *p* = 1.26e − 04) and macrophages infiltration (Cor = −0.406, *p* = 1.09e − 19), but a positive correlation with B cell infiltration (Cor = 0.357, *p* = 2.70e − 15), and dendritic cell infiltration (Cor = 0.226, *p* = 9.26e − 07) (Fig. 6J). Table 5 summarizes the correlation between differentially expressed interleukins and immune cell infiltration, highlighting the significant associations of CD8+ T cells (*p* = 0.022), IL11 (*p* = 0.014), IL23A (*p* = 0.005), and IL27 (*p* = 0.004) with RCC clinical outcomes.

**Table 5:** The cox proportional hazard model of interleukin and six tumor-infiltrating immune cells in RCC (TIMER)

	coef	HR	95%CI_l	95%CI_u	*p*-value	sig
B_cell	−1.361	0.256	0.01	6.423	0.408	
CD8_Tcell	−2.096	0.123	0.02	0.74	0.022	*
CD4_Tcell	−2.244	0.106	0.005	2.452	0.161	
Macrophage	−1.968	0.14	0.011	1.699	0.123	
Neutrophil	2.623	13.775	0.181	1045.726	0.235	
Dendritic	1.159	3.188	0.535	18.994	0.203	
IL1A	0.605	1.832	0.694	4.837	0.222	
IL4	3.522	33.849	0.365	3143.135	0.128	
IL7	−0.194	0.823	0.579	1.171	0.28	
IL11	0.41	1.507	1.087	2.09	0.014	*
IL15	−0.019	0.981	0.737	1.306	0.896	
IL16	−0.066	0.936	0.619	1.415	0.753	
IL23A	0.735	2.086	1.255	3.468	0.005	**
IL26	1.258	3.518	0.418	29.6	0.247	
IL27	0.969	2.636	1.365	5.089	0.004	**
IL32	0.097	1.101	0.943	1.287	0.224	

Note: Asterisk represent significant difference. *p < 0.05, **p < 0.01.

One study confirmed that intratumoral IL1A expression is an independent prognostic factor for ccRCC and that high IL1A expression is associated with shortened OS [5]. Analysis of the KM survival curve of IL1A using the TIMER database revealed that elevated IL1A expression significantly correlated with reduced cumulative survival time (*p* = 0.0137). This finding aligns with our earlier Kaplan-Meier Plotter analysis (Fig. 6K). Recent studies have reported that IL1A can rapidly induce neutrophil infiltration in rats with spinal cord injury (SCI). Additionally, IL1A is implicated in neutrophil recruitment. Therefore, in the Cox proportional hazards model, we performed a survival analysis of neutrophils *versus* IL1A, and the results showed that the cumulative survival time of KIRC patients with high IL1A expression and high neutrophil infiltration was significantly longer than that of KIRC patients with high IL1A expression and low neutrophil infiltration (*p* = 0.0017) (Fig. 6L).

### Differences in interleukin, interleukins at transcription factor targets, and kinase targets mRNA expression between HK-2 and Caki-1 cells

HK-2 cells served as the control group, while Caki-1 cells were designated as the experimental group. The expression levels of Interleukin mRNA were quantified using quantitative PCR (qPCR). The findings indicated notable upregulation in IL1A, IL7, IL15, IL16, IL26, IL27, and IL32, while IL11 and IL23A exhibited a marked downregulation (Fig. S4A). In comparison to HK-2 cells, Caki-1 cells exhibited significantly higher expression of LCK, LYN, and JAK3, but lower expression of SYK and FER, with all results being statistically significant (Fig. S4B).

### Differences in the expression of NFKB1 in HK-2 and Caki-1 cells and its regulatory role in interleukin

The mRNA expression of NFKB1 was significantly higher in Caki-1 cells compared to HK-2 cells (Fig. S4B). To investigate the regulatory role of NFKB1 on interleukins, Caki-1 cells were subjected to NFKB1 knockdown via siRNA transfection (Fig. S5A), The mRNA expression of IL7, IL15, IL16, IL23A, and IL32 were significantly upregulated, while IL1AmRNA expression was markedly downregulated. These findings indicate that NFKB1 negatively regulates IL7, IL15, IL16, IL23A, and IL32, while positively regulating IL1A (Fig. S5B).

### IL1A can promote neutrophil migration

**Figure 7:**
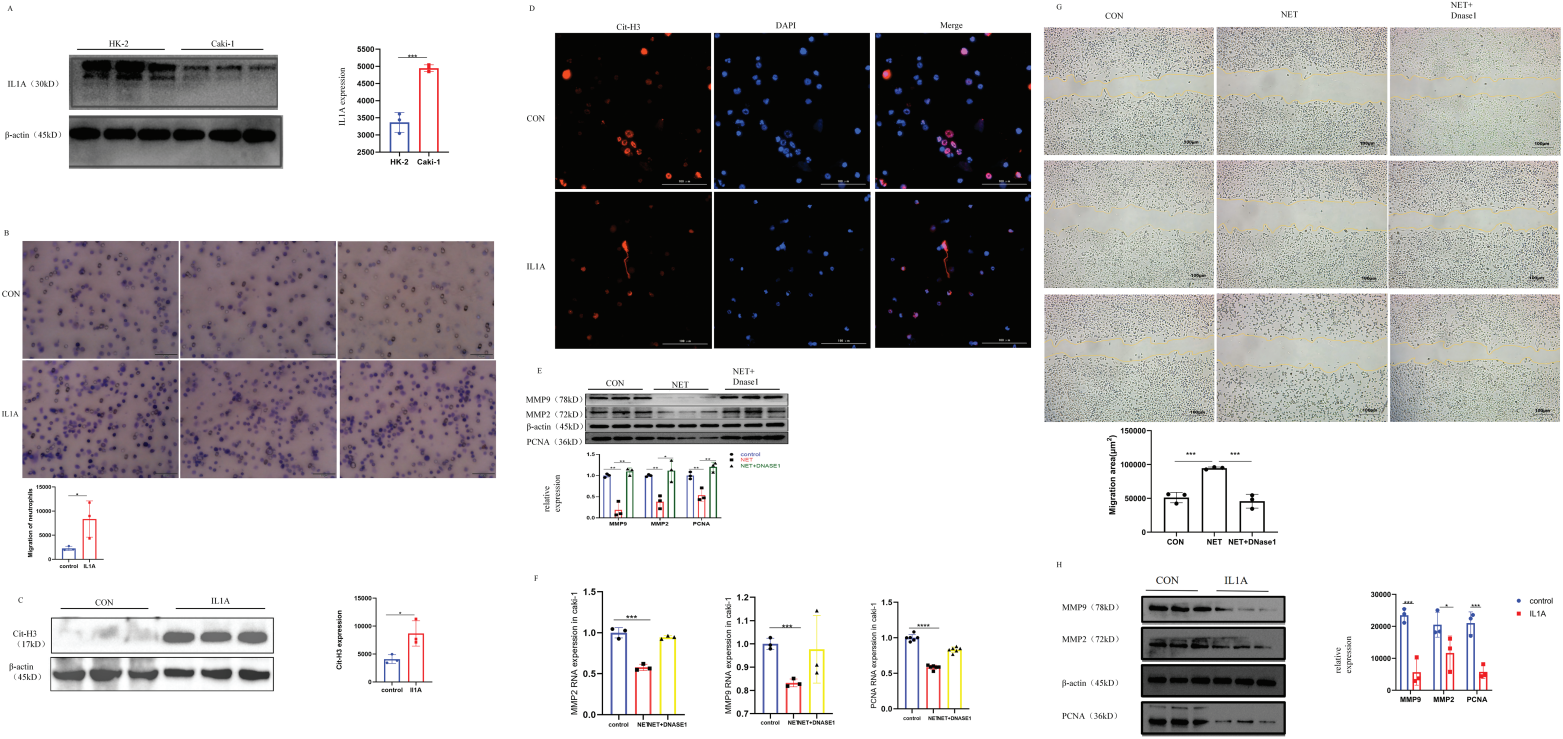
*In vivo* and *in vitro* experiments. (A) In Caki-1 cell line, the difference of IL1A protein expression was analyzed by Western Blot. Mean ± SEM, ***p < 0.001 *vs*. control or between the indicated groups, n = 3. Similar results were obtained from three independent culture assays. (B) The ability of neutrophils to migrate after IL1A addition was determined by transwell migration. Neutrophils were stained with crystal violet. Mean ± SEM, *p < 0.05 *vs*. control or between the indicated groups, n = 3. Similar results were obtained from three independent culture assays. (C) Western Blot was used to detect the effect of IL1A on the formation of NETs on neutrophils. Mean ± SEM, *p < 0.05, n = 3. (D) After co-culture of IL1A and neutrophils, there was a significant formation of NETs. Cit-H3 (red) and DNA (DAPI, blue) positioning suggest the existence of the NETs. (E) The protein expression levels of MMP2, MMP9, and PNCA in the NET group and NET + DNase1 group were detected by Western Blot. Mean ± SEM, *p < 0.05, **p < 0.01 *vs*. control or between the indicated groups, n = 3. Similar results were obtained from three independent culture assays. (F) The mRNA expression levels of MMP2, MMP9, and PNCA in the NET group and NET + DNase1 group were detected by q-PCR. Mean ± SEM, ***p < 0.001, ****p < 0.0001 *vs*. control or between the indicated groups, n = 3. Similar results were obtained from three independent culture assays. (G) The results of the cell scratch assay in Caki-1 cells showed that the percentage of wound healing distance between scratches of the NETs group was significantly higher than that of the control group. However, when DNase1 was added, the percentage of wound healing distance was comparable to the control group. Mean ± SEM, ***p < 0.001 *vs*. control or between the indicated groups, n = 3. Similar results were obtained from three independent culture assays. (H) The protein expression levels of MMP2, MMP9, and PNCA in the IL1A group and control group were detected by Western Blot. Mean ± SEM, *p < 0.05, ***p < 0.001 *vs*. control or between the indicated groups, n = 3. Similar results were obtained from three independent culture assays.

Cell migration plays a critical role in tumor progression, particularly in processes such as cancer metastasis and escape, and immune cell infiltration. Tumor infiltration by immune cells is highly regulated by both cancer cells and immune cells. Cancer cells utilize immunoediting within the tumor microenvironment to promote the recruitment of pro-tumor immune cells while suppressing anti-tumor immune cells. This process further enhances the metastatic potential of cancer cells. Western Blot analysis revealed a significantly higher expression of IL1A in Caki-1 cells compared to HK-2 cells (Fig. 7A). Caki-1 cells treated with IL1A served as the experimental group, while untreated Caki-1 cells were designated as the control group. The transwell migration assay revealed a significant increase in neutrophil migration in the experimental group compared to the control group (Fig. 7B). Our findings suggest that IL1A may promote neutrophil migration, potentially enhancing the infiltration of antineoplastic neutrophils and inhibiting the recruitment of pro-tumor neutrophils in KIRC therapy.

### IL1A induces the formation of NETs from neutrophils

NETs formation is now recognized as a distinct form of cell death. Unlike apoptosis, it occurs without caspase activation or phosphatidylserine exposure on the plasma membrane. Unlike necrosis, it involves extensive DNA release. NETs consist of DNA and a de-condensed chromatin scaffold that supports various proteins and enzymes including Myeloperoxidase (MPO), dependent on cell type. Histones are the most abundant proteins in NETs. Therefore, to explore whether IL1A could induce NETs formation from neutrophils, we employed immunofluorescence to visualize citrullinated histone H3 (Cit-H3) and western blot to quantify its protein expression. The results demonstrated NETs formation in neutrophils following IL1A treatment (Fig. 7CD). Our results provide preliminary evidence that IL1A induces NETs formation in neutrophils.

### NETs reduce the proliferation, migration, and invasion of Caki-1 cell

To further test the potential role of NETs on KIRC, we exposed Caki-1 cells to NETs. Western blotting was employed to assess the impact of NETs on Caki-1 cell proliferation, migration, and invasion. Our results showed that the expression of cell proliferation marker PCNA was significantly reduced after NETs treatment. Having established the effect of NETs on Caki-1 cell proliferation, we proceeded to evaluate their impact on migration and invasion through multiple experimental approaches. Previous studies indicate that Matrix Metalloproteinase (MMP) may contribute to cancer progression, invasion, and metastasis by degrading the extracellular matrix. This indicates that MMP2 and MMP9 may play pivotal roles in the invasion and metastasis of Caki-1 cells. Western blot (WB) and quantitative polymerase chain reaction (q-PCR) analyses revealed that NETs significantly reduced the expression of MMP2 and MMP9 (Fig. 7E,F). In addition, the wound healing assay demonstrated slower healing in the NETs group compared to the Control group (Fig. 7G). These results indicate that NETs expression negatively correlates with KIRC cell proliferation and significantly inhibits tumor migration and invasion. Based on bioinformatics analysis, we formulated a hypothesis that IL1A exerts a bidirectional influence on KIRC, depending on neutrophil infiltration levels. Western blot analysis of Caki-1 cells treated with IL1A showed significant downregulation of PCNA, MMP2, and MMP9 (Fig. 7H). This finding aligns with the previously observed effects of NETs on Caki-1 cells.

## Discussion

Interleukins (ILs) are a diverse group of cytokines with essential immunoregulatory roles. These cytokines are critical for regulating innate immune responses, promoting immune cell proliferation and differentiation, and mediating inflammatory cell recruitment. Notably, specific interleukins are essential for mediating inflammatory responses, immune cell proliferation, and differentiation. Extensive studies have shown that interleukins critically regulate tumor progression and exert immunotherapeutic effects within the tumor microenvironment [6,7]. Despite recent advancements, the prognostic significance and biological roles of interleukins in renal cell carcinoma (RCC) are not fully elucidated [3]. Therefore, additional research is necessary to clarify the intricate roles of interleukins in RCC and explore their therapeutic potential.

Mining tumor public databases are crucial for investigating and identifying the predictive role of interleukins in renal cell carcinoma. Currently, we utilize 10 distinct databases and online analysis tools. The screening of interleukins has significant potential for further clinical research. Among these, Oncomine stands as the world’s largest cancer microarray database, and provides extensive data on the cancer mutation spectrum, gene expression data, and relevant clinical information, facilitating the identification of novel biomarkers or therapeutic targets. UALCAN evaluates the epigenetic regulation of gene expression through promoter methylation and conducts PAN-CANCER gene expression analysis. GEPIA, an online analysis tool, calculates gene expression levels across multiple tumor types and investigates the genetic associations between gene expression and tumor prognosis. The Kaplan-Meier Plotter database conducts survival analysis across 21 tumor types and over 54,000 genes, offering a robust platform for identifying and validating survival biomarkers. The cBioPortal database serves as a comprehensive resource for tumor research, with a particular focus on TCGA data. It facilitates diverse analyses, including mutation profiling, data visualization, biological pathway exploration, survival analysis, and mutually exclusive genomic alteration analysis. GeneMANIA facilitates the prediction of gene and genome functions by identifying genes associated with input genes and uncovering novel genes with specific functions. The TRRUST database is a manually curated resource that documents transcription regulation networks, including target genes of transcription factors and their regulatory relationships. The STRING database enables the exploration of protein-protein interactions, encompassing both experimentally verified and computationally predicted interactions. Linked Omics provides access, analysis, and comparison of multi-omics data across various tumor types, supporting multi-omics association analysis. The TIMER database focuses on analyzing immune cell infiltration in tumor tissues, offering insights into potential therapeutic targets for renal cell carcinoma via correlation analysis and experimental validation. The Gene Expression Omnibus (GEO) database is a public bioinformatics repository that archives extensive gene expression data, encompassing microarray, RNA sequencing, protein mass spectrometry, and other biological experimental technologies.

In this study, we found that ten genes were differentially expressed in KIRC, with IL1A, IL4, IL7, IL15, IL16, IL23A, IL26, and IL32 being upregulated, and IL11 and IL27 being downregulated. The expression levels of IL11, IL23A, IL27, and IL32 were significantly correlated with tumor stage. Furthermore, IL1A, IL4, IL11, IL15, IL23A, and IL27 were significantly associated with better overall survival in KIRC patients. Although the GEO dataset analysis results partially diverged from the TCGA database findings, they collectively suggest a potentially significant role of interleukins in KIRC.

The accumulation of genetic alterations is a well-established driver of tumor progression. Herein, we hypothesize that the frequent genetic alterations in interleukins within KIRC synergistically and critically contribute to malignancy progression. To further elucidate the underlying mechanisms, we conducted GO and KEGG pathway enrichment analyses, revealing the significant roles of differentially expressed interleukins. These genes are primarily involved in cytokine-cytokine receptor interactions, natural killer (NK) cell-mediated cytotoxicity, and cell adhesion molecules. Notably, Feins and colleagues highlighted that chimeric antigen receptor (CAR) T cells represent a promising therapeutic approach for refractory lymphoid malignancies. Within this context, NK cells have emerged as a potential cell source for CAR-based therapies. Previous studies have demonstrated that cell-cell interactions and adhesion play critical roles in cancer progression. Our findings indicate that interleukins could serve as promising therapeutic targets for drug development in KIRC. Additionally, key transcription factors for interleukins have been identified, including homeobox A10 (HOXA10), v-myb myeloblastosis viral oncogene homolog (MYB), v-rel reticuloendotheliosis viral oncogene homolog A (RELA), nuclear factor of kappa light polypeptide gene enhancer in B-cells 1 (NFKB1) and Jun proto-oncogene (JUN) were identified. HOXA10 has been implicated in the deregulation of multiple cancer types via activation of the JAK1/STAT3 signaling pathway. Lu and Yarbrough reported that RELA phosphorylation contributes to cancer progression through regulating NF-κB signaling. NFKB1 acts as a suppressor of cancer progression by modulating the NF-κB-related pathway.

Furthermore, these transcription factors are enriched in cancer-related kinases such as lymphocyte-specific protein tyrosine kinase (LCK), LYN proto-oncogene (LYN), spleen tyrosine kinase (SYK), Janus kinase 3 (JAK3), and FER tyrosine kinase (FER). These kinases play a critical role in regulating tumor cell migration, invasion, and apoptosis. LCK, a member of the Src family kinases, enhances the efficacy of CAR T-cell therapy and the efficiency of CAR. LYN, another Src family kinase member, is associated with poor prognosis in renal cancer patients. Our results suggest that interleukins may regulate genomic stability, cell cycle progression, and the epithelial-mesenchymal transition (EMT) by influencing these kinases in KIRC.

Multiple studies have consistently demonstrated that immune cell infiltration is crucial in tumor progression and recurrence [8]. Our study further explores this complex relationship, uncovering a significant link between interleukin expression and the infiltration patterns of various immune cell subsets. These immune cells, including B cells, CD8+ T cells, CD4+ T cells, macrophages, neutrophils, and dendritic cells, are essential in the anti-tumor immune response. Our findings suggest that interleukins serve not only as prognostic markers, predicting the course of tumor development and recurrence, but also as indicators of the tumor microenvironment’s immune status.

Our study revealed that elevated IL1A expression is significantly correlated with poor prognosis in KIRC. Conversely, when high IL1A expression coexists with increased neutrophil infiltration, it is associated with a favorable prognosis. The role of IL1A in the tumor microenvironment is notably intricate [7,9]. It demonstrates anti-tumor activity through the activation of tumor-associated immune cells, including neutrophils [5]. On the other hand, high levels of IL1A have also been found to promote tumor cell growth, invasion, and migration [9]. In addition, the depletion of IL1A decreased the transcriptional activity of NF-κB, a key regulator of the majority of SASP components. Exogenous IL1A reinstated the capacity of senescent mouse fibroblasts to enhance prostate tumor growth through MTOR inhibition. This bidirectional regulatory mechanism highlights IL1A as a critical target in KIRC research. Our findings revealed that IL1A significantly suppressed the proliferation, migration, and invasion of Caki-1 cells.

Prior research has demonstrated that Dirty Necrosis (DN) in KIRC is characterized by extensive neutrophil infiltration and can serve as a potential adverse prognostic marker. It has recently been confirmed that DN in KIRC involves the formation and systemic validation of NETs. NETosis represents a specific form of neutrophil cell death. Numerous researchers have emphasized the impact of NETs on tumor progression and metastasis. Xiao et al. demonstrated that cathepsin C (CTSC), a tumor-secreted protease, promotes lung metastasis in breast cancer by recruiting neutrophils and inducing NET formation [10]. Neutrophils can exhibit either pro-tumor or anti-tumor functions, and the same applies to NETs [11]. NETs can inhibit the migration and proliferation of human melanoma cells cultured *in vitro*. NETs may also contribute to promoting anti-tumor immune responses [12]. For instance, in an *in vitro* co-culture model, NETs reduced the activation threshold of CD4+ T cells through upregulation of CD25 and CD69, as well as phosphorylation of ZAP70 [13]. Moreover, the injection of NET-DNA and NET proteins into subcutaneous tumors enhanced T lymphocyte recruitment, induced cancer cell death, and reduced tumor size [14]. Additionally, BCG, a treatment for bladder cancer, promotes NET formation, which correlates with the recruitment of T cells, monocytes, and macrophages to early-stage tumors, aiding in tumor control [15,16]. NETs were found to inhibit KIRC cell proliferation, migration, and invasion in our study.

Our study demonstrated that IL1A induces the formation of NETs, which may suppress KIRC cell proliferation, migration, and invasion, thereby exerting anti-tumor effects and influencing patient prognosis.IL1 signaling has been observed to impede mammary tumor growth and metastasis in luminal breast cancer. IL1A inhibits papilloma-to-carcinoma conversion in skin cancer and impedes cancer cell growth *in vitro* across multiple cancer types [7,9]. Additionally, several anticancer drugs that induce cancer cell senescence have been reported to elevate IL1 production [6,9]. IL1A is linked to a poor prognosis in lung adenocarcinoma (LUAD), unlike IL1B, indicating a potential role of IL1A in promoting LUAD tumor progression [7,17]. Hence, combining IL1A with other anti-tumor medications could present a novel approach to KIRC treatment.

Our study has demonstrated that IL1A exhibits potential anti-tumor effects on KIRC; however, interleukin monotherapy has encountered significant limitations in research endeavors over recent years. In a Phase I clinical trial, Bempegaldesleukin was assessed as a monotherapy in patients diagnosed with metastatic solid tumors, specifically melanoma and renal cell carcinoma [18]. In preclinical studies, CmAb-(IL10), a bispecific fusion protein, has demonstrated superior anti-tumor activity compared to IL10 fused with a non-tumor-specific antibody [19]. Gene therapy offers the potential for enhanced control over the localization and quantity of interleukins in therapeutic strategies. For instance, CBD-IL12, a fusion protein comprising IL12 and collagen binding domains, has demonstrated superior anti-tumor efficacy compared to IL12 alone [20]. Clinical trials have demonstrated the successful combination of IL36γ and IL23 with OX40 ligands, resulting in the promotion of inflammation and, consequently, effective tumor control [21]. Additionally, interleukins, including IL-2, IL-7, and IL-15, play pivotal roles in adoptive cell therapy (ACT) [13]. These cytokines have been extensively utilized to enhance the *in vitro* expansion and differentiation of adoptive cells, as well as to augment human ACT therapy through coadministration or genetic modification for infusion into transferred cells [22]. Notably, orthogonal IL-2-IL-2R pairs demonstrated efficacy with minimal toxicity in a murine melanoma model [23]. Recently, several clinical trials have embarked on utilizing IL12-expressing CAR T cells, aiming to mitigate uncontrolled IL12 release through stricter CAR T cell activation protocols, as compared to polyclonal tumor-infiltrating lymphocytes, and incorporating safety switches [24]. Tang et al. devised a cell-surface-conjugated nanogel capable of accommodating a substantial quantity of protein drugs [25]. This design mitigates IL15 toxicity by confining drug release specifically to the local tumor site where TCR activation occurs [25]. A recent preclinical investigation demonstrated that genetically modified CAR T cells incorporating the IL23 subunit p40 elicited autocrine IL23 signaling, thereby selectively promoting the proliferation of activated T cells and augmenting their antitumor efficacy [26]. An alternative hypothesis for the limited effectiveness of interleukin monotherapy posits that the administration of a solitary immune-finonstimulatory signal may be inadequate to elicit a sustained immune response capable of eradicating the tumor, thereby emphasizing the significance of combining interleukin with ACT therapy [27]. ACT demonstrates potent anti-tumor activity in hematologic malignancies, yet its efficacy in solid tumors remains limited [28]. Interleukins have the potential to enhance ACT by addressing its limitations in invasiveness and proliferation, as evidenced by the numerous clinical trials in this area, which underscore the promise of this approach. In summary, significant obstacles persist in the clinical application of interleukins, particularly IL1A, and the untapped potential of interleukin therapy to facilitate novel therapeutic avenues remains unexplored. Our study has the potential to benefit KIRC patients by addressing treatment resistance. Consequently, the direct clinical application of IL1A is significantly constrained. Additionally, modulators of IL1A could potentially be employed in conjunction with the recombinant engineering of the interleukin gene, as previously mentioned. Our research has revealed that NFKB1 positively modulates IL1A expression. Nevertheless, additional experiments are necessary to validate and elucidate the precise mechanism by which NFKB1 regulates IL1A.

The correlation between interleukins and KIRC was examined in our oncology database, with the chosen interleukins undergoing initial validation *in vitro*. Immunotherapy is a widely recognized and frequently utilized treatment option for patients with KIRC. A large-scale adjuvant KIRC trial has identified IL6 as a pivotal constituent within the tumor-enriched molecular signaling pathway [29]. Research has demonstrated that IL6 can suppress hepaCAM expression and stimulate KIRC cell proliferation via STAT3-mediated upregulation of either DNMT1 or DNMT3b [30]. RCC exhibits resistance to standard chemotherapy and radiotherapy; however, a complete response has been observed following high-dose interleukin-2 (IL-2) and interferon-alpha (IFN-α) immunotherapy [31]. The IL13 receptor α2 (IL13RA2) has been shown to suppress sunitinib-mediated apoptosis without enhancing tumor angiogenesis in the treatment of KIRC [32]. Consequently, the association between interleukin expression and KIRC holds promise as a novel prognostic therapeutic target and a potent adjunct for KIRC immunotherapy. However, this approach necessitates validation through extensive prospective studies. Furthermore, we offered insights into the potential mechanisms through which the identified IL1A could impact the prognosis of KIRC. Nevertheless, our study is not without limitations. Firstly, we have not experimentally validated the diagnostic and prognostic significance of interleukins aside from IL1A in KIRC. Secondly, while our study has demonstrated that IL1A can influence the prognosis of KIRC and holds potential as a biomarker for predicting clinical outcomes in KIRC patients, the small clinical sample size and uncertainties regarding its safety and clinical applicability necessitate further theoretical and practical investigations. Ultimately, given the significant heterogeneity of RCC, our study specifically evaluated the predictive potential of interleukin in KIRC, excluding other subtypes. Consequently, the predictive value of interleukin for other RCC subtypes remains unclear. Future studies using larger clinical tissue and serum samples are needed to further explore the complex interactions between interleukins and immune cells, with the goal of clarifying their roles in the diagnosis, progression, and prognosis of KIRC.

## Conclusion

Based on comprehensive bioinformatics analysis and preliminary experimental validation, our study has confirmed that IL1A potentially impacts the prognosis of kidney renal clear cell carcinoma (KIRC) patients by inducing neutrophil extracellular traps (NETs). These results offer new perspectives for identifying prognostic biomarkers in KIRC.

## Supplementary Materials

Figure S1 Analysis of ILs expression in KIRC. (A-F) In GSE6344 GSE15641, GSE16441 GSE16449, GSE53757 IL1A and GSE71963 data set analysis, IL4, IL7, IL11, IL15, IL16, IL23A, IL26, IL27 and IL32 differentially expressed. Nonsignificant results are not shown.

Figure S2 Analysis of the co-expression of kinase and TFs with ILs in KIRC and the correlation with OS. (A) mRNA expression of LCK, LYN, SYK, JAK3, and FER in RCC tissues and normal kidney tissues (UALCAN). (B) Relationship between LCK, LYN, SYK, JAK3, FER expression, and OS in RCC patients (Kaplan-Meier Plotter).

Figure S3 Differential expression of ILs-related genes in KIRC and the correlation with OS. (A) mRNA expression of HOXA10, MYB, NFKB1, and RELA in RCC tissues and normal kidney tissues (UALCAN). (B) Relationship between HOXA10, MYB, NFKB1, RELA expression, and OS in RCC patients (Kaplan-Meier Plotter).

Figure S4 Differential expression of ILs, kinase, and TFs in HK-2 and Caki-1 cell lines. (A) Differences in interleukin mRNA expression between HK-2 and Caki-1 cells.mRNA expression of IL1A, IL7, IL11, IL15, IL16, IL23A, IL26, IL27, IL32 in HK-2 cell and Caki-1 cells. (B) Differences in interleukins at transcription factor targets and kinase targets mRNA expression between HK-2 and Caki-1 cells. mRNA expression of LCK, LYN, SYK, JAK3, FER, NFKB1 in HK-2 cell and Caki-1 cells. ****p < 0.0001, ***p < 0.001, **p < 0.01, *p < 0.05

Figure S5 Alterations in ILs expression following NFKB1 SiRNA treatment. (A) Transfection efficiency of siRNA NFKB1 was measured after small interference of NFKB1 in Caki-1 cells. (B) Regulatory effect of NFKB1 on IL1A, IL7, IL15, IL16, IL23A, IL32. ****p < 0.0001.

## Data Availability

Not applicable.

## Abbreviations

RCC: Renal cell carcinoma
KIRC: Kidney renal clear cell carcinoma
IL: Interleukin
NETs: Neutrophil extracellular traps
TFs: Transcription factors
MPO: Myeloperoxidase
Cit-H3: Citrullinated Histone H3
MMP: Matrix metalloproteinase
NK: Natural killer
EMT: Epithelial-mesenchymal transition
DN: Dirty Necrosis
CAMs: Cell adhesion molecules
TCGA: The Cancer Genome Atlas
PPI: Protein–protein interaction
GO: Gene Ontology
KEGG: Kyoto Encyclopedia of Genes and Genomes
GPL: GEO Platform
BP: Biological processes
CC: Cellular components
MF: Molecular function
GSEA: Gene Set Enrichment Analysis
OS: Overall survival
RFS: Relapse-free survival
DSS: Disease-specific survival
PFS: Progression-free survival
DFS: Disease-free survival
LUAD: Lung adenocarcinoma
CTSC: Cathepsin C
HOXA10: Homeobox A10
MYB: v-myb myeloblastosis viral oncogene homolog (avian)
RELA: v-rel reticuloendotheliosis viral oncogene homolog A (avian)
JUN: JUN proto-oncogene
LCK: LCK proto-oncogene, Src family tyrosine kinase
LYN: LYN proto-oncogene, Src family tyrosine kinase
SYK: Spleen associated tyrosine kinase
JAK3: JANUS kinase 3
FER: FER tyrosine kinase
NFKB1: Nuclear factor of kappa light polypeptide gene enhancer in B-cells 1

## Supplementary Materials

The supplementary material is available online at https://doi.org/10.32604/or.2025.061978.
