# Supplementary figures and images for: IL1A: a novel prognostic biomarker and potential therapeutic target for renal clear cell carcinoma
Source: Oncol Res. 2025 Jun 26;33(7):1739–55. doi: 10.32604/or.2025.061978 (PMC12215607; doi:10.32604/or.2025.061978)

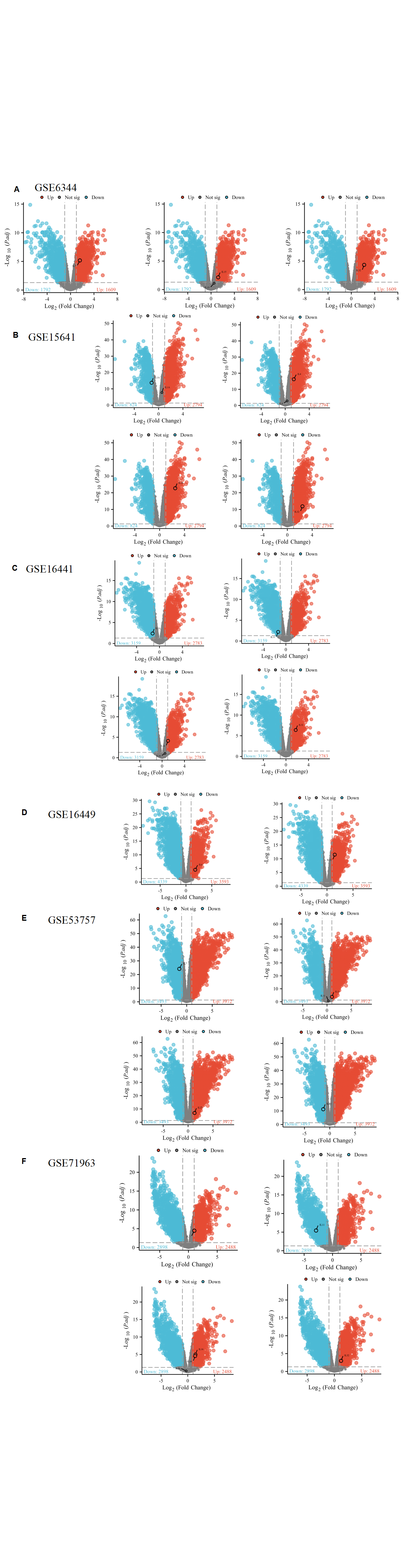

Supplement: Figure S1 [file OncolRes-33-61978-s001.tif]

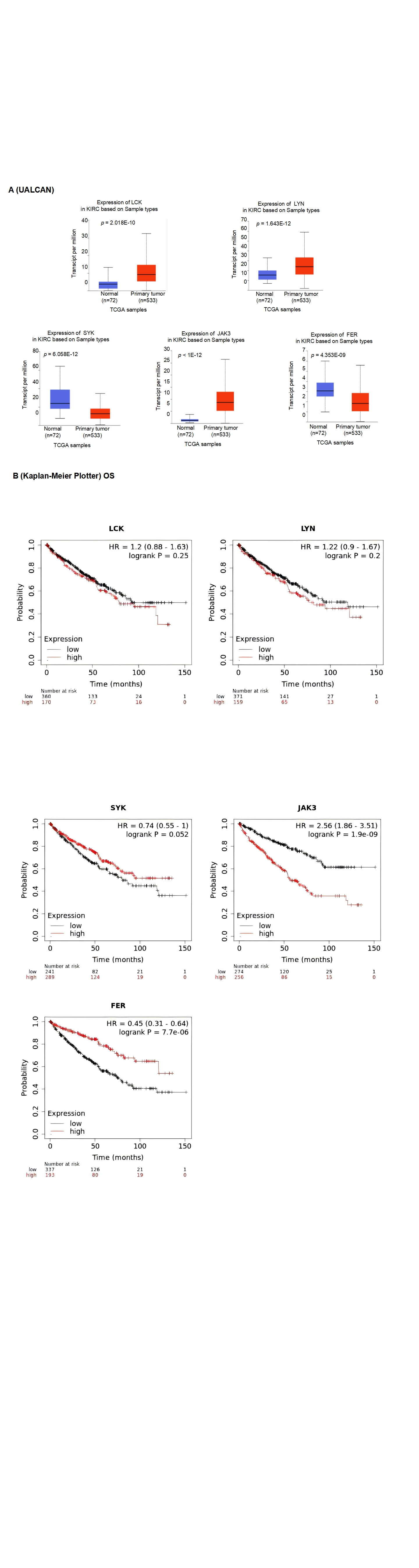

Supplement: Figure S2 [file OncolRes-33-61978-s002.tif]

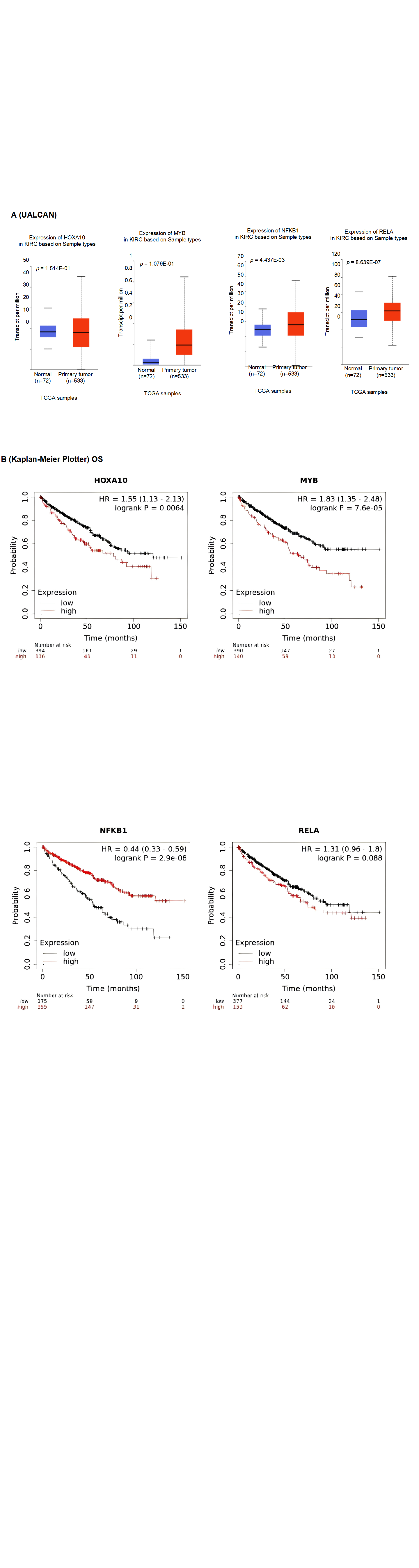

Supplement: Figure S3 [file OncolRes-33-61978-s003.tif]
